# EGFR alterations in glioblastoma play a role in antitumor immunity regulation

**DOI:** 10.3389/fonc.2023.1236246

**Published:** 2023-08-04

**Authors:** Xiao-Peng Li, Zheng-Qian Guo, Bao-Feng Wang, Min Zhao

**Affiliations:** Department of Neurosurgery, Tongji Hospital, Tongji Medical College, Huazhong University of Science and Technology, Wuhan, China

**Keywords:** glioblastoma, epidermal growth factor receptor, immunosuppression, tumor microenvironment, immune cell

## Abstract

The epidermal growth factor receptor (EGFR) is the most frequently altered gene in glioblastoma (GBM), which plays an important role in tumor development and anti-tumor immune response. While current molecular targeted therapies against the EGFR signaling pathway and its downstream key molecules have not demonstrated favorable clinical outcomes in GBM. Whereas tumor immunotherapies, especially immune checkpoint inhibitors, have shown durable antitumor responses in many cancers. However, the clinical efficacy is limited in patients carrying EGFR alterations, indicating that EGFR signaling may involve tumor immune response. Recent studies reveal that EGFR alterations not only promote GBM cell proliferation but also influence immune components in the tumor microenvironment (TME), leading to the recruitment of immunosuppressive cells (e.g., M2-like TAMs, MDSCs, and Tregs), and inhibition of T and NK cell activation. Moreover, EGFR alterations upregulate the expression of immunosuppressive molecules or cytokines (such as PD-L1, CD73, TGF-β). This review explores the role of EGFR alterations in establishing an immunosuppressive TME and hopes to provide a theoretical basis for combining targeted EGFR inhibitors with immunotherapy for GBM.

## 1 Introduction

**Figure 1:**
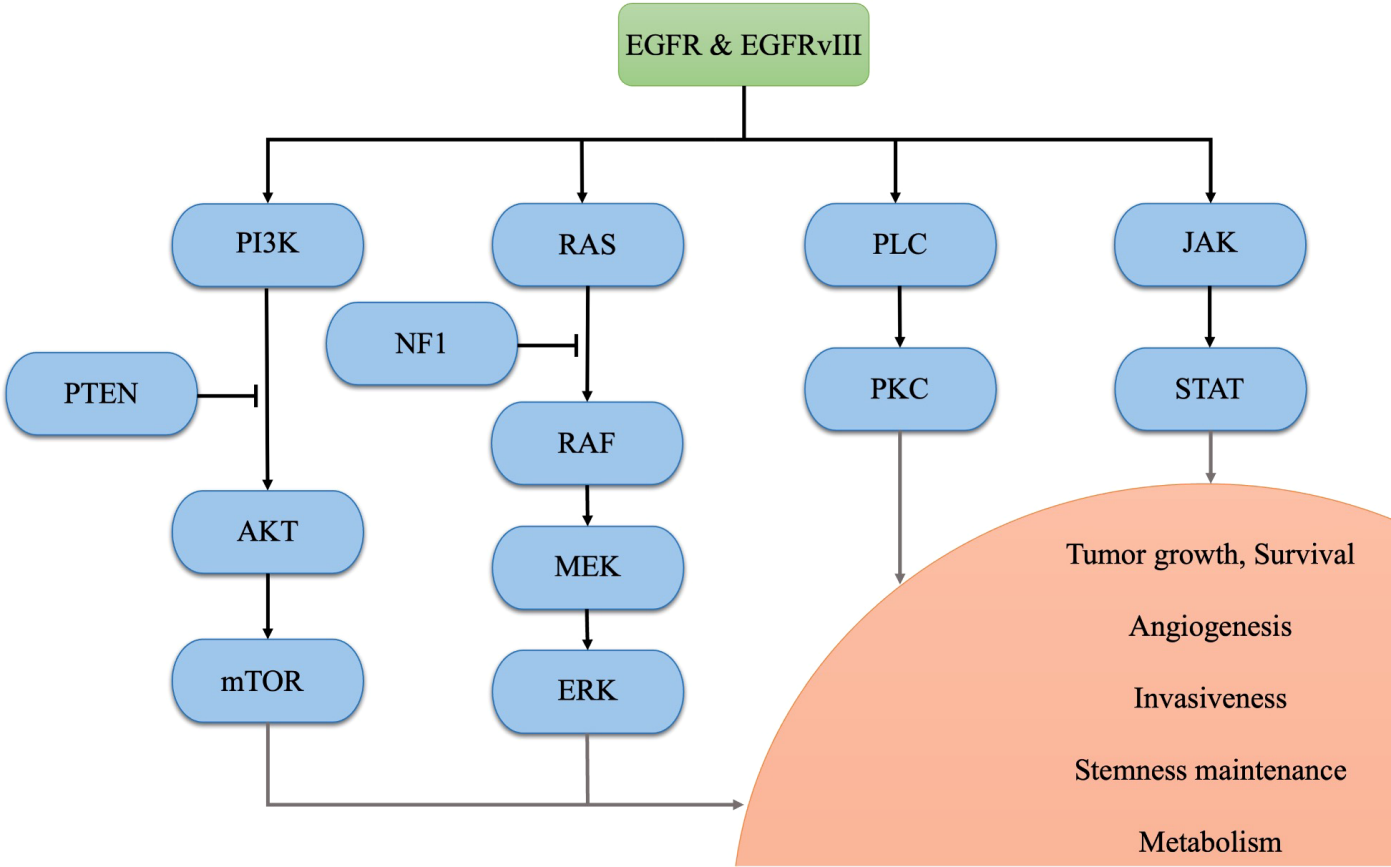
The identified mechanisms in the EGFR pathway in GBM.

Glioblastoma (GBM) is the most prevalent primary malignant tumor in the central nervous system (CNS) (1). The current standard treatment for newly diagnosed GBM involves maximal safe resection surgery, followed by temozolomide and adjuvant radiotherapy (2). Despite these interventions, only 6.9% of patients survive beyond five years post-diagnosis, based on the data from 2015 to 2019 in the United States (1). According to the 2021 World Health Organization (WHO) classification of tumors of the CNS, GBM is diagnosed based on the presence of necrosis, microvascular proliferation, and 1 or more of 3 specific genetic parameters [telomerase reverse transcriptase (TERT) promoter mutation, epidermal growth factor receptor (EGFR) gene amplification, combined gain of chromosome 7 and loss of chromosome 10 (+7/-10)] with wild-type forms of isocitrate dehydrogenase (IDH) 1/2 (3). Among these aberrances, EGFR is one of the most common oncogenic alterations (including gene amplification, mutation, rearrangement, and splicing site changes), occurring in approximately 50% of GBM samples (4, 5). After binding to ligands, EFGR forms a dimer that phosphorylates its C-terminal tail, regulating downstream physiological and pathological processes (5). Apart from this, EGFR variant III (EGFRvIII), a tumor-specific deletion of exons 2-7 which occurs in approximately 30% of GBM patients, consistently activates the EGFR signal even without ligands binding, contributing to intra-tumoral heterogeneity and resistance to targeted therapies (5–7). Aberrant activation of the EGFR and its downstream signaling pathways promotes GBM tumorigenesis and progression. Specifically, the phosphatidylinositol-3 kinase (PI3K)-AKT-mammalian target of rapamycin (mTOR) pathway and RAS-RAF-MEK-ERK pathway regulates tumor growth, survival, angiogenesis, and metabolism (7–9); the Janus kinase 2 (JAK2)-signal transducer and activator of transcription 3 (STAT3) pathway contributes to tumor growth, survival, stemness maintenance, and angiogenesis (7, 10), and the activated phospholipase C (PLC)-PKC pathway promotes tumor growth, survival, and invasiveness (11). Therefore, EGFR and its downstream signaling pathways are potential therapeutic targets for GBM. Notably, these pathways may contribute to establishing an immunosuppressive TME in EGFR-altered tumors (12–14). A bioinformatics study in glioma has revealed that EGFR mutation indicates poor prognosis and potential immune suppression within the TME (15). In terms of immunotherapy, EGFR amplification has been suggested as a biomarker of resistance to immune checkpoint inhibitors (ICIs) in GBM patients (16). In this review, we endeavor to compare the treatment targeted EGFR in GBM and discuss the mechanisms underlying the immunosuppressive TME regulated by the aberrant EGFR signaling pathway. The identified mechanisms in the EGFR pathway in GBM are illustrated in **Figure 1**.

## 2 Current strategies for targeting EGFR in GBM

Various treatments targeting EGFR have been explored for GBM. Three generations of EGFR tyrosine kinase inhibitors (TKIs) are approved for clinical use. First-generation TKIs (Erlotinib, Gefitinib, Lapatinib, etc.) inhibit the receptor by competitive binding with adenosine triphosphate (ATP) and second-generation TKIs (Afatinib, Dacomitinib, Tesevatinib) irreversibly inhibit all four ERBB receptors. Despite the preclinical data suggesting they affect tumor proliferation, all of them have poor responses in clinical trials on newly diagnosed and recurrent GBM due to insufficient delivery or resistance to inhibition (4–6, 17, 18). While a third-generation TKI, Osimertinib, is specifically designed to target the canonical EGFR activating mutations and the T790M resistance mutation with a good ability to cross the blood-brain barrier (BBB). It can overcome resistance to first- and second-generation TKIs by continuously blocking ERK signaling in GBM (4, 19), which may bring hope to GBM patients, but further clinical trials are needed to validate its potential. Novel methods, such as nanoparticles developed in preclinical animal models deliver and protect small interfering RNAs, allowing them to pass the BBB and knock down EGFR signaling, although their feasibility need to be verified before clinical application (20).

Immunotherapies targeting EGFR also face challenges in GBM. Rindopepimut, a vaccine against the EGFRvIII, prolonged progression-free survival (PFS) of patients with recurrent GBM but failed to increase overall survival (OS) in phase III clinical trial (21). In addition, EGFRvIII chimeric antigen receptor (CAR) T therapy has been tested in GBM. The engineered lymphocytes were detectable in both peripheral blood and tumor samples from patients after CAR T infusion, but no satisfactory results were obtained in phase I study and there was one treatment-related death due to the severe toxicity (22). ICIs in preclinical GBM mouse models confirmed the safety and efficiency of monoclonal antibodies targeting the PD-1/PD-L1 axis (23), but have little clinical benefit in GBM, especially in patients harboring EGFR amplification. A recent clinical study showed that after initiation of PD-1 inhibitor (pembrolizumab or nivolumab) for recurrent GBM patients with EGFR amplification, the median OS was 7 months compared to 18 months for those without EGFR amplification (16).

Despite achieving encouraging progress in various cancers, these treatments have not achieved the expected effectiveness in GBM patients with EGFR alterations. Factors such as complex regulatory networks, high heterogeneity of tumor cells, BBB blockage, and altered TME contribute the less therapeutic improvements against GBM (24). On the other hand, both the EGFR targeting therapy and immunotherapy demonstrate favorable results in preclinical models but fail in clinical trials, suggesting that mice still have significant limitations in modeling human cancer and immunity.

## 3 EGFR signaling in GBM regulates the infiltration of immune cells

The TME infiltrates various non-cancerous cells, including astrocytes, pericytes, endothelial cells, fibroblasts, and immune cells. It has long been considered that the normal brain is one of the “immune privileged” organs (25). However, the disruption of the BBB in GBM leads to immune cells infiltrating to the tumor mass from blood flow. Immunosuppressive cells include tumor-associated microglia and macrophages (TAMs), myeloid-derived suppressor cells (MDSCs), regulatory T cells (Tregs), and regulatory B cells (Bregs), while the anti-tumor immune cells include T cells (CD4+ helper T cells, CD8+ cytotoxic T cells) and natural killer (NK) cells, etc. Infiltration or dysfunction of these cells is one of the causes of the highly immunosuppressive and “cold” TME phenotype of the GBM (26).

### 3.1 Microglia and myeloid-derived cells

TAMs, comprising microglia and macrophages, are the dominant infiltrating immune cells in GBM and account for 30-50% of the tumor mass (27, 28). Microglia are yolk sac–derived and have limited self-renewal capacity, while macrophages are monocyte-derived from the bone marrow and peripheral circulation and constituted approximately 85% of the total TAM population (29, 30). Despite different origins, they both play a vital role in tumor progression. The activated TAMs in GBM can be simply divided into tumor-suppressing M1-like phenotype and tumor-promoting M2-like phenotype with great plasticity and heterogeneity. The main TAMs in GBM present possess an M2-like phenotype, promoting tumor invasion, proliferation, angiogenesis, and immune evasion through the expression and secretion of matrix-degrading enzymes, angiogenic factors, and immunosuppressive cytokines/chemokines (28, 31). However, it should be noted that dichotomously classified M1 and M2 phenotypes were fitted well *in vitro* under optimal conditions, as it does not fully reflect the complexity of TAMs activation. Additional states (such as M2a, M2b, M2c, and M2d states) have also been identified, suggesting that TAMs *in vivo* likely have more functions along the M1/M2 spectrum. Several studies have shown that EGFR alterations associated with TAMs infiltration in GBM, and inhibiting EGFR by pharmacy strongly decreased microglia-stimulated invasion in GL261 GBM cells (32). Another study using GBM cell lines U87 and A172 *in vitro* and *in vivo* shows that EGFR cooperates with EGFRvIII to induce macrophage infiltration by upregulation of chemokine C-C motif ligand 2 (CCL2) through the KRAS pathway, one of the major downstream pathways of the EGFR (33). In addition, dual targeting of EGFR and mTOR pathways has also been demonstrated to inhibit tumor growth and macrophage infiltration on GBM xenografts by downregulation of CCL2 (34), an immunosuppressive cytokine that shapes the macrophage polarization toward a tumor-promoting, immunosuppressive phenotype, and significantly shortened the survival of GBM-bearing mice (30, 35). Furthermore, a recent single-cell RNA sequencing study focused on the immune landscape during GBM progression found that subsets of pro-inflammatory microglia in developing GBMs, while anti-inflammatory macrophages are observed in end-stage tumors. This evolution parallels the extensive growth of EGFR+ GBM cells (36). Another single-cell level study reveals that GBM samples with EGFR and cyclin-dependent kinase 4 (CDK4) co-amplifications in the same cell exhibit higher infiltration of CD163+ immunosuppressive macrophages (37).

MDSCs are a heterogeneous population of immature bone marrow cells consisting of two cell subsets: granulocytic or polymorphonuclear MDSCs (PMN-MDSCs) and monocytic MDSCs (M-MDSCs). MDSCs exert their immunosuppressive effects in GBM by inhibiting cytotoxic T cell activity, suppressing the function of NK cells, macrophages, and dendritic cells, and augmenting the effect of Tregs (38, 39). The density of MDSCs also correlates with tumor stages, chemotherapy response, and patient prognosis in GBM (39). According to two recent studies using GBM animal models, it has been observed that, similar to TAMs, MDSCs accumulation is more pronounced in EGFR (+) GBM, when compared to the EGFR-wild type (EGFR-WT) (36, 40). These studies also found that EGFRvIII GBM highly expressed chemokine C-X-C motif ligand 1/2/3 (CXCL1/2/3) and PMN-MDSCs-expressed chemokine C-X-C motif receptor 2 (CXCR2) constitute an axis that regulates the output of PMN-MDSCs from the bone marrow, leading to increased systemic levels of these cells. Interestingly, pharmacological inhibition of this axis benefitted for response to ICIs and prolonged survival in EGFRvIII-driven GBM mice (40). In summary, the immunosuppressive TME of EGFRvIII GBM may be due to the greater infiltration by MDSCs, targeting this cell is a potential therapeutic strategy for GBM, but further research is needed in this area.

### 3.2 Tumor-infiltrating lymphocytes

TILs are a group of tumor-infiltrating and antigenic cell populations that exist in TME, and their amount and subtypes determine the clinical outcomes and treatment responses in glioma patients (41, 42). CD4+ helper T cells stimulate an anti-tumor response by activating CD8+ cytotoxic T cells and promoting B cell proliferation and differentiation. CD8+ cytotoxic T cells induce tumor apoptosis through T cell receptor activation or lyse tumor cells by releasing interferon-γ, perforin, and granzyme (43). However, T cells are rare in the GBM TME, accounting for less than 0.25% of total isolated cells from GBM biopsies. The majority of samples exhibit a “lymphocyte depletion” phenotype with few CD8+ cytotoxic T cells but increased infiltration of Tregs, particularly in cases with EGFR amplification (44, 45). On the other hand, dysfunction of T cells (such as senescence, tolerance, anergy, exhaustion, and ignorance) is a hallmark of GBM, leading to ineffective anti-tumor immune responses (28, 43, 46). It has been suggested that the efficacy of EGFR-TKIs depends mainly on the presence of CD4+ and CD8+ T cells, as treatment significantly enhances the immune responses against the tumor (47). In contrast to conventional CD4+ and CD8+ T cells, the FOXP3+Tregs, a subset of CD4+ T cells, play an opposite role in the GBM microenvironment. Tregs inhibit the activation of effective T cells through the interactions with cytotoxic T lymphocyte antigen-4 (CTLA-4) and CD80/86 on antigen-presenting cells (APCs). They also secrete immunosuppressive cytokines that promote tumor progression and polarization of macrophages toward an M2-like phenotype (48). How the aberrant EGFR signaling pathway regulates these T cells in the GBM microenvironment may be related to the expression of some immunosuppressive molecules or cytokines, such as programmed death-ligand 1 (PD-L1) (23), extracellular-5’-nucleotidase (CD73) (49), and transforming growth factor (TGF)-β (50), which will be discussed in detail in the next section.

NK cells are innate lymphoid cells that account for a small proportion of tumor-infiltrating cells, which exert anti-tumor effects through lytic granules secretion and recruiting other immune cells (51). However, some GBM patients show reduced NK cell activation due to decreased levels of the natural killer group 2 member D (NKG2D) receptor on the NK cell surface (28). Additionally, NKG2D ligands in cancer cells correlated positively with the activation of EGFR and its downstream MEK pathway, which is commonly hyperactivated in those tumors and reduced by EGFR inhibitors (52). However, different NKG2D ligands can function as target molecules for NK cell-mediated immunosurveillance or tumor immune escape. NKG2D ligands expressed on the cell surface of tumor cells can be recognized by NK cells through NKG2D and promote a cytotoxic response that leads to tumor cell elimination (53). However, soluble NKG2D ligands generated by a disintegrin and metalloproteinase 10 (ADAM10), ADAM17, and matrix metalloproteinase 14 (MMP14), can promote NKG2D down-regulation, impairing NK cell-effector functions and facilitating tumor immune escape (54, 55). Therefore, to determine the mechanism of EGFR signaling pathway regulation of NK cells in GBM, further studies are needed to focus on whether GBM with EGFR alterations express higher levels of NKG2D ligands or which form of the ligands are expressed in EGFR-altered GBM.

Tumor infiltrating B cells also play a role in shaping tumor development. Activated B cells display antitumor activity by producing immunoglobulins, promoting T cell responses, and killing tumor cells directly. Other B cells with tumor-promoting effects are defined as Bregs, upregulate PD-L1, CTLA-4 and secrete immunosuppressive cytokines such as IL-10 and TGF-β, attenuating the response of T and NK cells while facilitating the activation of Tregs, MDSCs, and TAMs (56). However, no relevant studies have yet shown the association between altered EGFR signaling pathways in tumor cells and the infiltration and activation of Bregs, especially in GBM. Further investigations are needed to explore this field.

## 4 Immunosuppressive molecules and cytokines regulated by EFGR signaling in GBM

GBM cells also express cell surface molecules or secrete various chemokines, cytokines, and growth factors that regulate immune cell infiltration or function in GBM. Some of them are modulated by EGFR and its downstream signaling.

### 4.1 PD-L1

The expression of PD-L1 in GBM correlates with the patient prognosis (57, 58). However, the PD-L1 positivity rate in GBM specimens ranges from 61.0% to 88% in different studies (59). Such inconsistent results might be associated with different PD-L1 detection techniques, tumor heterogeneity, and different specimen sources. PD-L1 binding to PD-1 on T cells restrains the anti-tumor response, inducing apoptosis or anergy in activated T cells and promoting the infiltration of Tregs, leading to tumor immune escape (23). Recent studies have found that PD-L1 expression in GBM cells is associated with EGFR and its downstream signaling pathways (23). Among them, EGFR-dependent PI3K activation, phosphatase and tensin homolog deleted on chromosome ten (PTEN) loss, and AKT activation induce the β-catenin binding to the CD274 gene (encode PD-L1) promoter region, resulting in increased PD-L1 expression (60, 61). Another study found that the activated EGFR-ERK signaling pathway in GBM upregulates COP9 signalosome subunit 6 (CSN6) and PD-L1 protein expression, stabilizing PD-L1 and inhibiting its degradation (62). Radiotherapy has also been shown to increase PD-L1 expression in glioma cells. In a vitro study, irradiated human GBM cell lines U87 and U251 show significant upregulation of PD-L1 expression at the protein and mRNA levels via phosphorylation of EGFR and its downstream signaling molecule JAK2 (63). A single-center retrospective study has found that GBM patients with EGFR amplification exhibit shorter median OS after receiving ICIs (16). Therefore, targeting EGFR and its downstream signaling, in combination with PD-1/PD-L1 ICIs, may hold the potential in restoring the T-lymphocyte killing capacity and improving the sensitivity to immunotherapy and targeted therapy in tumor patients (47, 64, 65).

### 4.2 CD73/adenosine

CD73 is anchored to cell membrane lipid rafts and highly expressed in various tumors including GBM and plays a role in immune regulation by generating adenosine in the TME. Adenosine is a component of adenine nucleotides that regulates immune cell function by the ectonucleoside triphosphate diphosphohydrolase (NTPDase1, CD39)-CD73 synergic effect in glioma TME (66). ATP and adenosine diphosphate (ADP) released from tumor cells are hydrolyzed by CD39 to adenosine monophosphate (AMP), which is then further dephosphorylated to adenosine by CD73 (67, 68). In cancer patients, alterations in adenosine deaminase (ADA) activity can lead to increased levels of adenosine as ADA normally deactivates adenosine by converting it to inosine (68). Adenosine mediates its regulatory functions by binding to adenosine receptors (A2AR and A2BR) on the tumor-infiltrating immune cells, triggering the accumulation of intracellular cyclic adenosine monophosphate (cAMP). This signaling molecule is associated with the establishment of an immunosuppressive TME characterized by the polarization of TAMs into a tumor-promoting M2-like phenotype, increased infiltration and immunosuppressive activity of Tregs and MDSCs, and suppressed activity of dendritic cells, CD8+ T cells, and NK cells (67–69). Overexpression of CD73 promotes immunosuppression and is associated with poor prognosis in multiple cancers (66, 70). A Recent *in vivo* study has revealed that blocking CD73 expression in the tumor cells can potentially regulate the GBM immune microenvironment and inhibit tumor growth by inducing apoptosis (71). Another study based on single-cell RNA sequencing found a correlation between high CD73 expression and EFGR amplification as well as hypoxia in GBM (49). These findings suggest that EGFR alterations signaling shape an immunosuppressive TME in GBM by promoting CD73 expression. Targeting the CD73-adenosine axis may hold promise as a therapeutic strategy in combination with EGFR-TKIs.

### 4.3 TGF-β

TGF-β is a pro-tumorigenic cytokine overexpressed and produced by various cells in GBM, contributes to tumor growth, angiogenesis, maintenance of glioma stem cell stemness, and the establishment of an immunosuppressive TME (72, 73). TGF-β exerts its antitumor immune response on immune cells within the TME. For innate immunity, TGF-β promotes TAMs recruitment and M2-like polarization, monocyte differentiation into MDSCs, and attenuating the tumor killing efficiency of NK cells. For adaptive immunity, it facilitates Tregs persistence, inhibiting the function of effector T cells and antigen-presenting dendritic cells (12, 50, 74). In addition, high-level TGF-β also contributes to poor response to PD-L1 immune therapy (75). Recent studies have revealed that the activation of TGF-β may be related to EGFR singling and its downstream molecules. The activation PLCγ-PKC pathway leads to intragin-αvβ activation and contributes to local TGF-β activate from its latent to its bioactive form (12, 76). Although it is well known that the correlation between EGFR signaling and TGF-β expression in many cancers. Further studies specific to GBM are needed to verify this finding.

Overall, EGFR and its downstream signaling pathways modulate immune-related molecules and cytokines in GBM, influencing the immune cell landscape and establishing an immunosuppressive TME. Targeting these interactions may hold promise for improving the effectiveness of immunotherapy and targeted therapy in GBM treatment.

## 5 The roles of EGFR in immune cells

A variety of immune cells express EGFR, which plays a significant role in the TME of tumors without EGFR alterations. Recently, some studies have identified the function of EGFR in macrophages and T cells. In both human and mouse tumors (hepatocellular carcinoma and colorectal carcinoma), EGFR expression in macrophages promotes tumor development (77, 78). Myeloid-specific EGFR knockout mice exhibited less carcinogenesis, possibly associated with decreased IL-6 production via the STAT3 pathway. This suggests a pro-tumorigenic role of EGFR signaling in myeloid cells (77, 78). Similarly, in a colitis-associated carcinogenesis model, EGFR signaling in macrophages plays a critical role in tumor development by activating macrophages and inducing polarization toward a tumor-promoting M2-like phenotype (79). EGFR expression has also been detected in Tregs. Amphiregulin (AREG), an EGF-like growth factor derived from mast cells, enhances the suppressive function of Tregs by activating the MAPK pathway through binding to EGFR on Tregs (80). In the central nervous system, EGFR phosphorylation and subsequent activation of ERK mediate microglia migration during inflammatory states. This process may be mediated by the lipopolysaccharide (LPS)-triggered intracellular calcium mobilization. And the calcium activity is decisive for EGFR phosphorylation initiated by its ligands (81). All these cells play key roles in the immunosuppressive microenvironment of GBM, which predicts that targeting the EGFR signaling pathway and its downstream key molecules in immune cells may be a new therapeutic strategy that should be explored in future studies.

## 6 Conclusion

**Figure 2:**
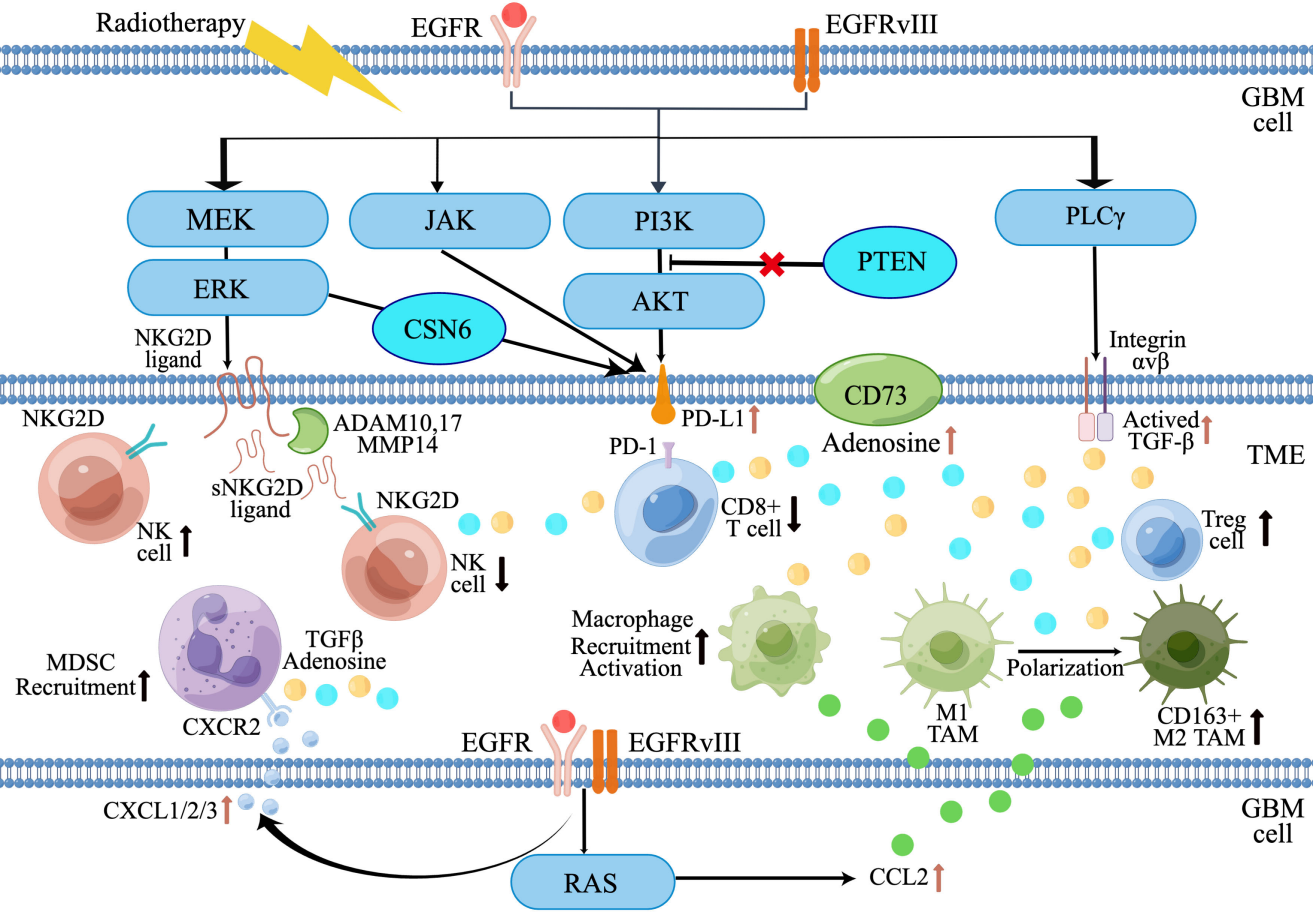
Diagram of EGFR alterations in GBM that promote an immunosuppressive tumor microenvironment. (By Figdraw).

Aberrant EGFR signaling pathways in GBM contribute to tumor progression by directly promoting tumor cell proliferation and survival, as well as establishing an immunosuppressive TME that allows for tumor immune escape. The EGFR signaling pathway recruits TAMs and MDSCs, converting M1-like TAMs to tumor-promoting M2-like phenotype, which further induce T cell and NK cell dysfunction. EGFR alterations in GBM also contribute to the upregulation of immunosuppressive molecules and cytokines, such as PD-L1, CD73, TGF-β, etc. (**Figure 2**). It also should be noted that the downstream signaling described in this review is very similar to other receptor tyrosine kinases (RTKs), EGFR-targeted therapies face the challenge of resistance due to the upregulation of redundant RTKs and activation of compensatory signaling pathways. Understanding how these redundant RTKs modulate the TME in GBM requires further investigation. On the other hand, to enhance the efficacy of immunotherapy in GBM patients with EGFR alteration, it is necessary to develop new therapeutic strategies targeting the immunosuppression TME associated with EGFR alterations. Future approaches could involve combining molecularly targeted therapies and immunotherapy to stimulate both the immunogenic response of GBM and the anti-tumor immune response. For example, the combination of a PD-L1 inhibitor (Atezolizumab) and EGFR-TKI (Erlotinib) has shown promising prospects in EGFR-mutant non-small cell cancer (82, 83). Implementing a similar integrated approach in GBM could improve treatment outcomes and benefit a larger number of patients.

## Author contributions

X-PL and Z-QG wrote the manuscript and drew the figures. B-FW and MZ conceived the main outline took charge of manuscript revision in English. All authors contributed to the article and approved the submitted version.
